# Tumor-agnostic ctDNA levels by mFAST-SeqS in first-line HR-positive, HER2 negative metastatic breast cancer patients as a biomarker for survival

**DOI:** 10.1038/s41523-023-00563-w

**Published:** 2023-07-14

**Authors:** Noortje Verschoor, Vanja de Weerd, Mai N. Van, Jaco Kraan, Marcel Smid, Joan B. Heijns, Jan C. Drooger, Johanna M. Zuetenhorst, Annemieke van der Padt-Pruijsten, Agnes Jager, Stefan Sleijfer, John W. M. Martens, Saskia M. Wilting

**Affiliations:** 1 Department of Medical Oncology, Erasmus MC Cancer Institute, Rotterdam, The Netherlands; 2 Department of Medical Oncology, Amphia, Breda, The Netherlands; 3 Department of Medical Oncology, Breast Cancer Center South Holland South, Ikazia Hospital, Rotterdam, The Netherlands; 4 Department of Medical Oncology, Franciscus Gasthuis & Vlietland, Rotterdam/ Schiedam, the Netherlands; 5 Department of Internal Medicine, Breast Cancer Center South Holland, Maasstad Hospital, Rotterdam, The Netherlands

**Keywords:** Breast cancer, Tumour biomarkers, Prognostic markers, Breast cancer, Cancer genetics

## Abstract

This prospective cohort study reports aneuploidy score by mFast-SeqS as a strong prognostic marker in MBC patients. mFAST-SeqS is an affordable and easily implementable method for the assessment of total ctDNA levels and, as such, provides an alternative prognostic tool. One mixed cohort (cohort A, *n* = 45) starting any type of treatment in any line of therapy and one larger cohort (cohort B, *n* = 129) consisting of patients starting aromatase inhibitors (AI) as first-line therapy were used. mFAST-SeqS was performed using plasma of blood in which CTCs (CellSearch) were enumerated. The resulting aneuploidy score was correlated with categorized CTC count and associated with outcome. The aneuploidy score was significantly correlated with CTC count, but discordance was observed in 31.6% when applying cut-offs of 5. In both cohorts, aneuploidy score was a significant prognostic marker for both PFS and OS. In the Cox regression models, the HR for aneuploidy score for PFS was 2.52 (95% CI: 1.56–4.07), and the HR for OS was 2.37 (95% CI: 1.36–4.14). Results presented here warrant further investigations into the clinical utility of this marker in MBC patients.

## Introduction

Circulating tumor cells (CTCs) have been extensively researched as noninvasive markers of tumor load in metastatic breast cancer^1^. CTCs can be quantified by the frequently used CellSearch method (Menarini Silicon Biosystems Inc.). The prevalence of CTCs is approximately 60% in metastatic breast cancer (MBC) patients. In about 50% of patients, CTCs are above the validated cut-off value of 5 in 7.5 ml blood, thereby indicating aggressive disease^1–3^. For breast cancer, there is the level I evidence of CTC count above this cut-off value for being prognostic for progression-free survival (PFS) and overall survival (OS), independent of receptor subgroup and therapy line^4,5^. However, the clinical utility of CTC enumeration has not been proven yet to this date^6,7^. The enumeration of CTCs requires specialized equipment and trained staff, which could hamper clinical implementation.

Alternatively, circulating tumor DNA (ctDNA) can be used to estimate tumor load in the blood. Previous work showed the prognostic value of the levels of ctDNA in MBC patients, but a high level of evidence has not yet been achieved^8^. ctDNA level is often expressed as the variant allele frequency (VAF) of the dominant tumor-derived mutation, determined by next-generation sequencing (NGS) panels. Such panels report on mutations and copy number variations in a number of known cancer-associated genes and thus provide valuable, yet restricted information about the interrogated tumor genome. In contrast to tumor-informed approaches like targeted NGS, approaches such as whole genome sequencing or whole exome sequencing are used to obtain more extensive information on tumor load, as well as on possible targets for treatment and predictive biomarkers. However, most assays are rather expensive and need considerable bioinformatical support to interpret their results^9,10^. Furthermore, applying these methods requires a relatively high cfDNA input, mostly found only in patients with late-stage disease^11^. Moreover, most of the above-mentioned methods have a long turn-around time, prohibiting their use as biomarkers of tumor load in daily clinical practice.

In contrast, the modified fast aneuploidy screening test-sequencing system (mFAST-SeqS) represents a fast and fairly affordable assay to estimate the fraction of ctDNA by assessing chromosomal aneuploidy instead of mutations. Since in the majority of malignancies, aneuploidy is a hallmark, this method is readily applicable in multiple tumor types and is, therefore, tumor-agnostic^12^. Because it is an amplicon-based NGS method, it only requires standard equipment present at virtually any molecular and/or diagnostics laboratory. The method was initially designed to pre-screen plasma samples for a high ctDNA content to aid further analysis with methods that require high input, for example, complete or shallow whole-genome sequencing. In that setting, it has been previously shown that an aneuploidy score of five or higher derived by mFAST-SeqS had a sensitivity of 100% and a specificity of 80% for the presence of copy number aberrations revealed by shallow whole-genome sequencing^13^. Moreover, aneuploidy score of five or higher is strongly associated with a mutation allele frequency of 5–10% or higher, assessed by targeted sequencing^14^.

In a proof-of-concept study by Suppan et al., it has subsequently been shown that the aneuploidy score by itself had prognostic value in MBC^15^. Thus, this blood-based aneuploidy score might be a relevant clinical biomarker. Notwithstanding the promising results, this pilot study was performed in a small group of 29 MBC patients of different receptor subtypes and in different lines of treatment. The prognostic value for aneuploidy score is yet to be validated for MBC patients. Additionally, it is currently not yet fully elucidated to what extent CTC count and levels of ctDNA correlate.

In the present work, we investigate the correlation between CTC count and the ctDNA-load, estimated by the aneuploidy score derived by mFAST-SeqS. Secondly, we aim to assess the clinical validity of the aneuploidy score by analyzing the prognostic value for survival in two separate MBC cohorts.

## Results

### Description of samples

A total of 92 and 157 patients were included in cohorts A and B, respectively. From cohort A, blood draws that were not taken at the start of a new line of therapy (*n* = 31), and non-unique patient samples (*n* = 14) were excluded. From cohort B, ctDNA isolation could not be performed in twelve samples because there was no sufficient amount of plasma available. As a result, correlation analysis between aneuploidy and CTC count was conducted in 45 and 145 patients, respectively. For assessment of prognostic value, patients from cohort B that did not start a first-line aromatase inhibitor (*n* = 10) and patients that started an aromatase inhibitor because of poor clinical performance (*n* = 6) were excluded, upon which 129 patients remained (flowchart in supplementary figure 1). The baseline characteristics of both cohorts were different, mainly due to the inclusion criteria (Table 1). The median follow-up in cohort A was 11 months (95% CI: 1–37 months), in which 40 patients experienced progressive disease and 30 patients died. In cohort B, the median follow-up time was 27 months (95% CI: 2–73 months), in which 81 patients had progressive disease, and 58 patients died. The most important difference between the two cohorts was that patients in cohort A were unselected for the receptor subtype. Furthermore, 37 patients (82.2%) were treated with one or multiple lines of therapy before starting a new line of therapy, as opposed to cohort B, which consisted only of patients that were sampled before the start of first-line aromatase inhibitors. The more advanced stage of disease of patients in cohort A is reflected in a greater proportion of patients with a high CTC count (*p* = 0.02, chi-square) and with a high aneuploidy score, although this was not significantly different (*p* = 0.13, chi-square).

**Table 1:** Baseline characteristics of cohort A (*n* = 45) and cohort B (*n* = 129).

Characteristic	Cohort A (*n* = 45)	Cohort B (*n* = 129)
*Age*		
Mean (range) (yr)	56 (33–79)	68 (40–89)
*WHO PS at registration—no. (%)*		
0	18 (40.0)	41 (31.8)
1	17 (37.8)	74 (57.4)
2–3	5 (11.1)	14 (10.9)
Unknown	5 (11.1)	0
*Subtype (%)*		
HR+	30 (66.7)	129 (100)
HER2+	9 (20.0)	0
TNBC	6 (13.3)	0
*Disease-free interval (months)*		
Mean ± SD	49 ± 61	182 ± 220
Visceral disease—no. (%)	31 (68.9)	63 (48.8)
*Previous treatment lines (%)*		
0	8 (17.8)	129 (100)
1–2	20 (44.4)	0
>2	17 (37.8)	0
*Treatment after blood draw*		
Endocrine therapy	15 (33.3)	129 (100)
* Addition of CDK4/6i*	*6 (13.3)*	*36 (27.9)*
Chemotherapy	25 (55.6)	0
Targeted therapy	5 (11.1)	0
*Baseline CA15.3*		
Median (IQR)	69 (232)	77 (279)
<30 (%)	12 (36.4)	21 (22.1)
≥30 (%)	21 (63.6)	74 (77.9)
Missing	12	34
*CTC count*		
Median (IQR)	9 (187)	3 (20)
<5 (%)	18 (40.0)	76 (58.9)
≥5 (%)	27 (60.0)	53 (41.1)
*Aneuploidy score*		
Median (IQR)	4.2 (24.3)	3.6 (5.1)
<5 (%)	24 (53.3)	83 (64.3)
≥5 (%)	21 (46.7)	46 (35.7)
*Survival status*		
Alive	15 (33.3)	71 (55.0)
Breast cancer-related death	29 (64.4)	42 (32.6)
Not breast cancer-related death	0	5 (3.9)
Unknown	1 (2.2)	11 (8.5)

### Comparison between biomarkers

**Fig. 1:**
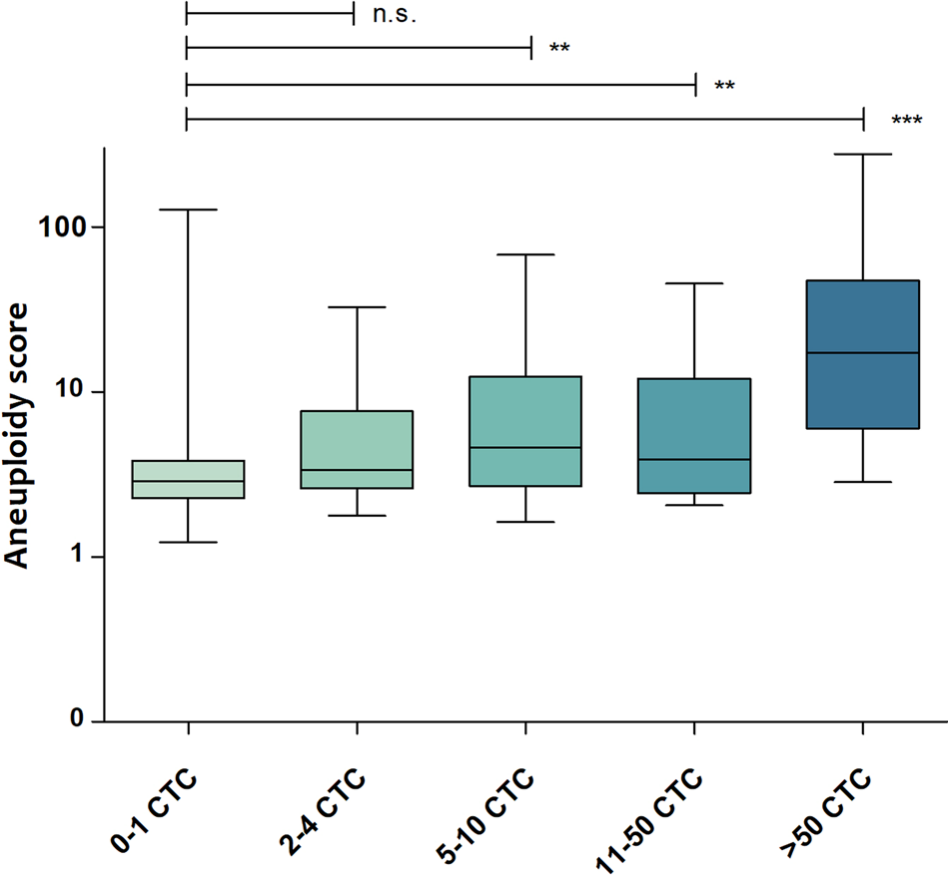
Association between aneuploidy score and CTC count. Boxplots showing the association between classes of circulating tumor cell counts and median aneuploidy score (Jonckheere–Terpstra test for trend *p* < 0.001). The boxes indicate the 25th to 75th percentile, the middle line indicates the median, and the whiskers represent minimal and maximal values. The lines above the graph show interclass comparisons by two-sided Mann–Whitney *U* tests: n.s. = non significant, ** = *p* < 0.001, *** = *p* < 0.0001.

In all samples combined (*n* = 190), there was a significant correlation between aneuploidy score and CTC count (Spearman’s rho 0.49, *p* < 0.01). Additionally, there was a significant trend for a higher median aneuploidy score with increasing CTC count (Jonkcheere–Terpstra test *p* < 0.001, Fig. 1). Seventy-three patients (41.9%) had low scores on aneuploidy score and CTC count (both <5), whereas 46 patients (26.4%) had high scores (≥5) on both markers (correlation plots shown in supplementary figure 2). There was a significant association between a high aneuploidy score and a high CTC count (chi-square test, *p* < 0.001). However, discordancy was also observed in 55 patients, of which 34 (19.5%) only had a high CTC count, and 21 (12.1%) only had a high aneuploidy score. The aneuploidy score was not different between patients with and without the visceral disease (Mann–Whitney test, *p* = 0.54), nor between patients with liver metastases and with metastases at other sites (Mann–Whitney test, *p* = 0.08). Additionally, the baseline CA15.3 measurement was available for 128 patients, of whom 74% showed levels above the clinically used cut-off of 30. There was no correlation between CA15.3 and aneuploidy score (Spearman Rho 0.06, *p* = 0.50). When considering samples with an aneuploidy score of 5 or higher, alterations in chromosomes 1q and 8q (copy number gain) as well as chromosomes 8p, 10q, 13q, and 17p (copy number loss, Supplementary Fig. 3) were most frequently observed. Lastly, supplementary figure 4 demonstrates a significant correlation (Spearman’s rho 0.66, *p* < 0.001) between VAF and aneuploidy score in patients with a detectable mutation by the Oncomine™ Breast cfDNA panel (*n* = 24).

### Association of aneuploidy score with survival measures

**Fig. 2:**
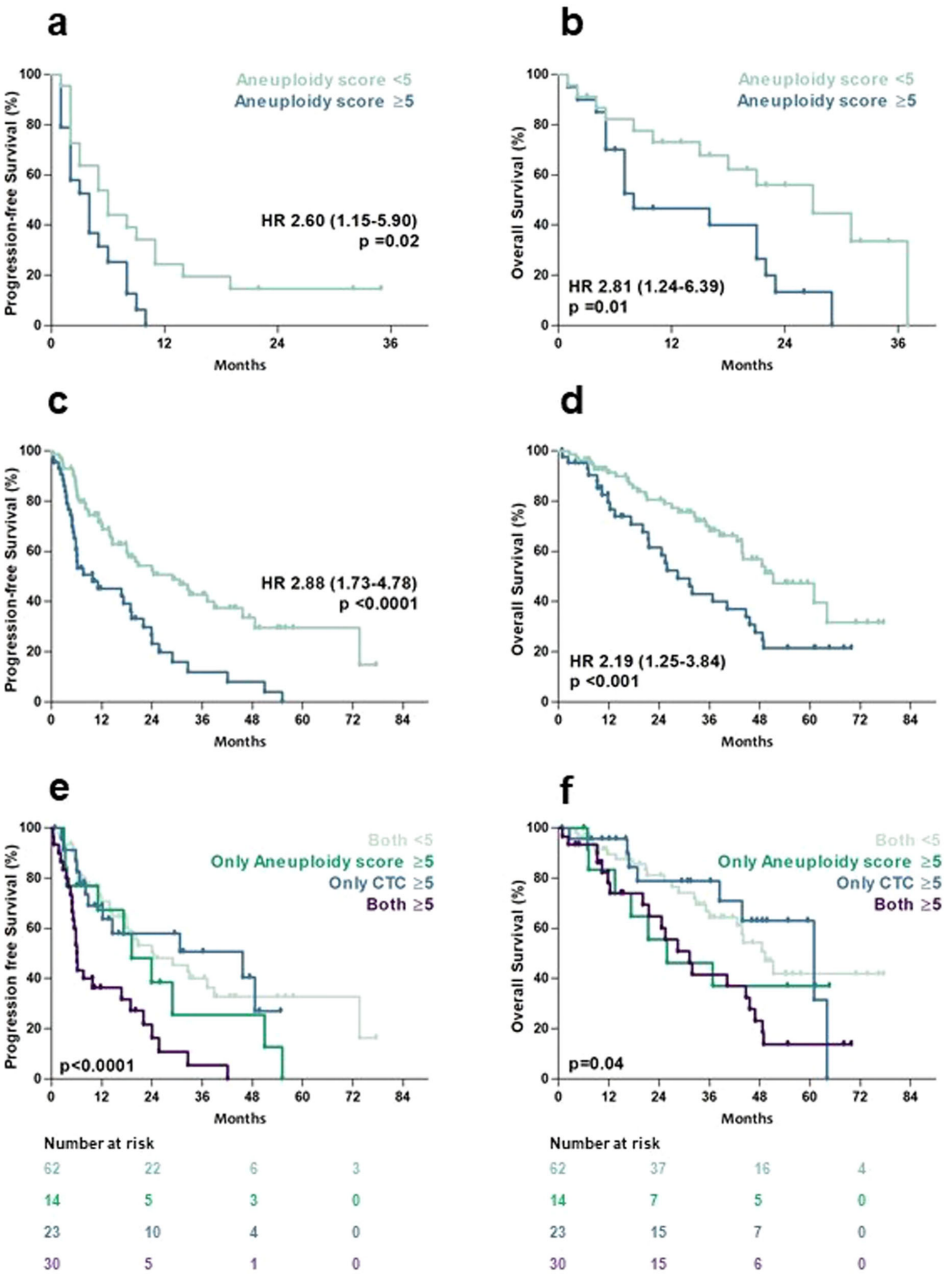
Kaplan–Meier estimates of probabilities of survival. Progression-free survival (panel **a**) and overall survival (panel **b**) in cohort A and of progression-free survival (panel **c**) and overall survival (panel **d**) in cohort B. Panel **e** and **f** show Kaplan–Meier estimates of probabilities of progression-free survival and overall survival for aneuploidy score combined with CTC count in cohort B, with an associated number at risk tables below. All p-values were derived from two-sided log-rank tests.

Next, the potential prognostic value of an elevated aneuploidy score was evaluated. The Kaplan–Meier curves in Fig. 2a–d show that a high aneuploidy score was significantly associated with PFS and with OS in cohort A as well as in cohort B (all *p* < 0.05, log-rank test). When combining both aneuploidy and CTC count in cohort B, patients that had both an elevated aneuploidy score and an elevated CTC count had the shortest PFS (6.0 months, 95% CI: 5.3–6.8 months) and OS (31.2 months, 95% CI: 21.8–40.7 months). Patients with a high aneuploidy score and a low CTC count had comparable OS to the patients with both markers elevated, whereas patients with elevated CTC count and a low aneuploidy score did not (median 25.9 and 60.9 months, respectively, log-rank test for trend *p* = 0.04, Fig. 2e). For PFS, this effect was not observed, although patients with both markers elevated clearly had worse outcomes on endocrine therapy (log-rank test for trend *p* < 0.0001, Fig. 2f). Multivariable Cox regression with PFS as outcome measure demonstrated that a high aneuploidy score was a significant prognostic marker for PFS with a hazard ratio of 2.52 (95% CI: 1.56–4.07), independent of visceral disease, while the CTC count was not significantly associated. For OS, the hazard ratio in the multivariable model for a high aneuploidy score was 2.37 (95% CI: 1.36–4.14), next to significant associations for WHO performance 2 status and the presence of visceral disease (Table 2).

**Table 2:** **A**. Univariate and multivariate Cox regression analysis for PFS in cohort B. **B**. Univariate and multivariate Cox regression analysis for OS in cohort B.

	Univariate		Multivariate	
Characteristic	HR (95% CI)	*p*-Value	HR (95% CI)	*p*-Value
**A**				
Age	0.99 (0.97–1.01)	0.18		
*WHO PS (ref 0)*				
1	1.26 (0.78–2.05)	0.35		
2	0.75 (0.28–1.97)	0.56		
Disease free interval	1.00 (1.00–1.00)	0.68		
Visceral disease (Y/N)	1.49 (0.96–2.32)	0.07	1.75 (1.12–2.75)	0.02
CDK4/6i (Y/N)	0.71 (0.42–1.21)	0.21		
CA15.3 ≥ 30 (Y/N)	1.01 (0.53–1.91)	0.98		
Aneuploidy score ≥ 5 (Y/N)	2.45 (1.57–3.82)	<0.001	2.52 (1.56–4.07)	<0.001
CTC-count ≥ 5 (Y/N)	1.69 (1.08–2.63)	0.02	1.33 (0.83–2.11)	0.24
**B**				
Age	1.02 (0.98–1.05)	0.09	1.02 (0.99–1.05)	0.15
*WHO PS (ref 0)*				
1	1.95 (1.02–3.74)	0.04	1.54 (0.78–3.04)	0.22
2	3.95 (1.53–10.15)	0.004	3.43 (1.17–10.0)	0.03
Disease free interval	1.00 (1.00–1.00)	0.46		
Visceral disease (Y/N)	2.08 (1.23–3.53)	0.007	2.74 (1.56–4.79)	<0.001
CDK4/6i (Y/N)	0.96 (0.52–1.75)	0.88		
CA15.3 ≥ 30 (Y/N)	0.92 (0.40–2.12)	0.85		
Aneuploidy score ≥ 5 (Y/N)	2.03 (1.21–3.40)	0.007	2.37 (1.36–4.14)	0.002
CTC count ≥ 5 (Y/N)	1.41 (0.84–2.36)	0.20		

*P*-values indicate a significant difference in hazard ratio compared to the reference cohort.

## Discussion

In the present work, we established the independent prognostic value of the aneuploidy score for PFS and OS in MBC patients. First, we validated the findings of Suppan et al. by showing that aneuploidy score correlated with outcome. Next, we further emphasized these findings in a larger, homogeneous cohort of patients that were treated with first-line aromatase inhibitors. Finally, multivariable analysis showed that the aneuploidy score was prognostic, independent of other common clinical determinants.

As expected, the aneuploidy score correlated with CTC count, as both represent circulating tumor-specific markers of cancer cell turnover. The copy number profiles that we found on the chromosome arm level support the tumor-specificity of our mFast-SeqS results since these are in line with what was reported before in breast cancer^16^. It was remarkable that CTC count did not prove to be a significant prognostic factor in our cohort, which is contradictory to previous literature^4^. In the analysis by Bidard et al., only 14% of patients started with endocrine treatment in different lines of treatment. Patients starting with aromatase inhibitors as first-line treatment have a relatively indolent disease, as also demonstrated by the lower median CTC count in our cohort. Thus, this specific patient group might have been underrepresented in the previously presented pooled analysis, and a different cut-off might be more suitable.

Important limitations of the mFast-SeqS include the limited sensitivity compared to the assessment of mutations and the low resolution. Furthermore, since this method is aimed at detecting aneuploidy, it cannot be used to detect targetable alterations, for example, mutations in PIK3CA. It is already known from the literature that measuring copy number alterations in circulating DNA harbors a higher detection limit than mutations or structural rearrangements because the analysis is based on increased or decreased numbers of reads of a specific segment, whereas the DNA sequence itself has not changed from healthy DNA^14,17^. In addition, due to the LINE-1-based amplicons, specific (focal) copy number alterations cannot always be measured. For example, it is not possible to assess HER2 amplification status by this method due to the fact that there are no LINE-1 elements on the HER2 amplicon. However, one might hypothesize that for establishing a prognosis in metastatic disease, a very sensitive assay is not always required. The benefits of the aneuploidy score, being the easy implementation and interpretation, the low costs, and its usefulness in multiple tumor types, might outweigh these disadvantages in this context.

A limitation of the current analysis is that the cut-off of five that we used for the aneuploidy score was not established for association with outcome, although this threshold was used in other cohort studies for this purpose as well^18,19^. Our aim was to validate the prognostic value that was previously reported for MBC patients. However, it requires a larger, independent cohort to certify the most appropriate threshold. Further analysis with more patient data will help with finding the optimal, reliable cut-off before assessing the possible clinical utility of the aneuploidy score.

The major strength of our analysis includes the two independent cohorts that we used to validate the previous findings, one of which consisted of a well-annotated cohort of patients all about to start the same first-line endocrine therapy. We showed that baseline CA15.3, which is currently used as a biomarker in the clinic, does not correlate to the aneuploidy score nor to the outcome. However, this marker is used in a more longitudinal fashion in clinical practice, and we did not collect serial samples. That the aneuploidy score could serve as an early response marker of treatment was shown by Suppan et al., but our cohorts are not suited to validate this finding.

Collectively, our study demonstrates that mFAST-SeqS is a promising method to assess tumor load without the need for prior knowledge of the mutational landscape. It is widely applicable because it requires no specialist equipment or computationally intensive bioinformatical pipelines. Furthermore, it is affordable. Lastly, since the DNA of virtually all solid tumors harbors aneuploidy to some extent and because low input of only 1 ng cfDNA is required, this method is applicable in multiple metastatic settings. Further research should first determine the most appropriate cut-off and the added value of serial sampling, and next, whether there is clinical utility in treatment stratification of MBC patients by aneuploidy score. In an era with increasing treatment possibilities and increasing health care costs, the applicability of the aneuploidy score might lie in identifying those patients in need of escalating therapy.

## Methods

### Patient inclusion

Between May 2015 and September 2021, patients with MBC were consecutively included in one of the two following prospective cohort studies, which were approved by the Medical Research Ethics Committee of the Erasmus MC Cancer Institute (Rotterdam, the Netherlands): in cohort A (Liquid Biobank, Erasmus MC ID MEC 17–238) patients in any line of treatment and with any clinical subtype were included when there was a clinical need for CTC enumeration. In cohort B (Caremore-AI, Erasmus MC ID MEC 14–588), patients with hormone-receptor, HER2 negative MBC that was about to start treatment with first-line aromatase inhibitors (AI) ± CDK4/6 inhibitors were prospectively included. Exclusion criteria were adjuvant chemotherapy within 6 months, other anticancer therapy within two weeks, hormonal antitumor treatment within 1 week prior to the start of AI, and medical conditions prohibiting adequate follow-up. All patients provided written informed consent for the data presented here.

### Blood samples and assays

Blood was drawn in CellSave preservative tubes. CTCs were enumerated in 7.5 mL peripheral blood with the CellSearch® Circulating Epithelial Cell Kit within 96 h, according to the manufacturer’s instructions. The remaining blood was centrifuged at 1700×*g* for 10 min, and plasma was transferred to a new tube. Then a second centrifugation step was performed at 12,000×*g* for 10 min at 4 °C. Plasma was stored at −80 °C until further processing. For cfDNA isolation, 0.3–1.6 mL plasma was isolated using the Maxwell® (MX) RSC LV ccfDNA Plasma Custom Kit (Promega, Madison, WI, USA) for cohort A and the QIAamp Circulating Nucleic Acid Kit (Qiagen) for cohort B and quantified by the Quant-iT dsSNA High-sensitivity Assay (Invitrogen, Life Technologies) according to the manufacturer’s instructions. The Qubit Fluorometer (Invitrogen) was used as a readout. cfDNA was then stored at −20 °C. Aneuploidy score was obtained by mFAST-SeqS essentially as previously described^19^. Briefly, 1 ng of cfDNA was amplified using a Phusion high fidelity polymerase (New England Biolabs) by a single primary primer pair for specific amplification of LINE-1 sequences throughout the genome (2′ at 98 °C, followed by 8 cycles of 10” at 98 °C, 2′ at 57 °C and 2′ at 72 °C). To increase the complexity of the resulting sequencing libraries, a random spacer was introduced to the forward primer as described by Fadrosh et al.^20^ (see Supplementary Table 1 for primer sequences). By a second PCR step (2′ at 98 °C, followed by 18 cycles of 10” at 98 °C, 15” at 65 °C and 15” at 72 °C), indexes and adapters were added using Illumina index primer sequences coupled to the common sequence in the LINE-1 primers (Supplementary Table 1). The resulting libraries were pooled equimolarly and sequenced on a MiSeq system generating 150 bp single-end reads to reach at least 90,000 reads per sample. Read counts per chromosome arm were normalized to the total library size, and subsequently, a *Z*-score per chromosome arm was calculated relative to healthy female controls. The short arms of chromosomes 13, 14, 15, 21, and 22, as well as chromosome Y, were excluded due to the insufficient presence of LINE-1 elements. Finally, the resulting *Z*-scores were squared and summed and compared to genome-wide squared and summed values of the controls, yielding an overall aneuploidy score, which indicates the number of standard deviations the sample deviating from the healthy controls.

### Statistical considerations

The correlation between CTC count and aneuploidy score was calculated by Spearman’s correlation coefficient and the Jonckheere–Terpstra test for trends across ordered groups. Additionally, to account for the fact that the CTC count can be zero, the proportion of patients with a high aneuploidy score (≥5) was compared between groups with a low CTC count (CTC < 5) and a high CTC count (CTC ≥ 5) with a chi-square test. In a small subgroup of patients, the VAF of the driver mutation by the Oncomine™ Breast cfDNA Assay was previously described^21^. The correlation between VAF and aneuploidy score was calculated by Spearman. Next, for both cohorts, survival differences were explored and visualized with the Kaplan-Meier method and compared univariate using the log-rank test for PFS and OS. Cohort A was too small to correct for known prognostic factors. Therefore, multivariable analysis was performed with Cox proportional-hazards regression in cohort B only, thereby eliminating the possible effect of hormone and HER2 receptor subtype and line of treatment. The following factors were considered: age, disease-free interval (both as continuous variables), presence of visceral metastases, the concurrent start of CDK4/6 inhibitors, baseline CA15.3, CTC-count, and aneuploidy score (all as dichotomous variables, CTC count and aneuploidy score were scored low or high according to the previously established cut-off of five). Factors with a *p*-value of <0.1 at univariable analysis were included in the multivariable model using backward stepwise selection. This lenient significance level was used to prevent the false rejection of borderline significant variables. *P*-values < 0.05 in the multivariable model were considered significant. Survival was defined from the date of blood draw to radiological or clinical progression (for PFS) or death (for OS). If this did not occur, patients were censored at the date of last contact. All statistical analyses were performed using IBM SPSS Statistics v25 (IBM Corp., Armonk, NY, USA), and for all tests, two-sided *p*-values of <0.05 were considered statistically significant. Figures were generated with GraphPad Prism 5 and Biorender. Results were reported according to the REMARK guidelines^22^.

### Reporting summary

Further information on research design is available in the Nature Research Reporting Summary linked to this article.

## Supplementary information

Supplementary information

Reporting summary

## Data Availability

The data that support the findings of this study are available from the corresponding author upon reasonable request.

## Code availability

Code for mFast-Seqs processing can be obtained from the corresponding author upon reasonable request.
