# Supplementary material for: Tumor-agnostic ctDNA levels by mFAST-SeqS in first-line HR-positive, HER2 negative metastatic breast cancer patients as a biomarker for survival
Source: NPJ Breast Cancer. 2023 Jul 14;9:61. doi: 10.1038/s41523-023-00563-w (PMC10349058; doi:10.1038/s41523-023-00563-w)
Supplement: Supplementary file 1 — Supplementary information [file 41523_2023_563_MOESM1_ESM.pdf]

Supplementary Information

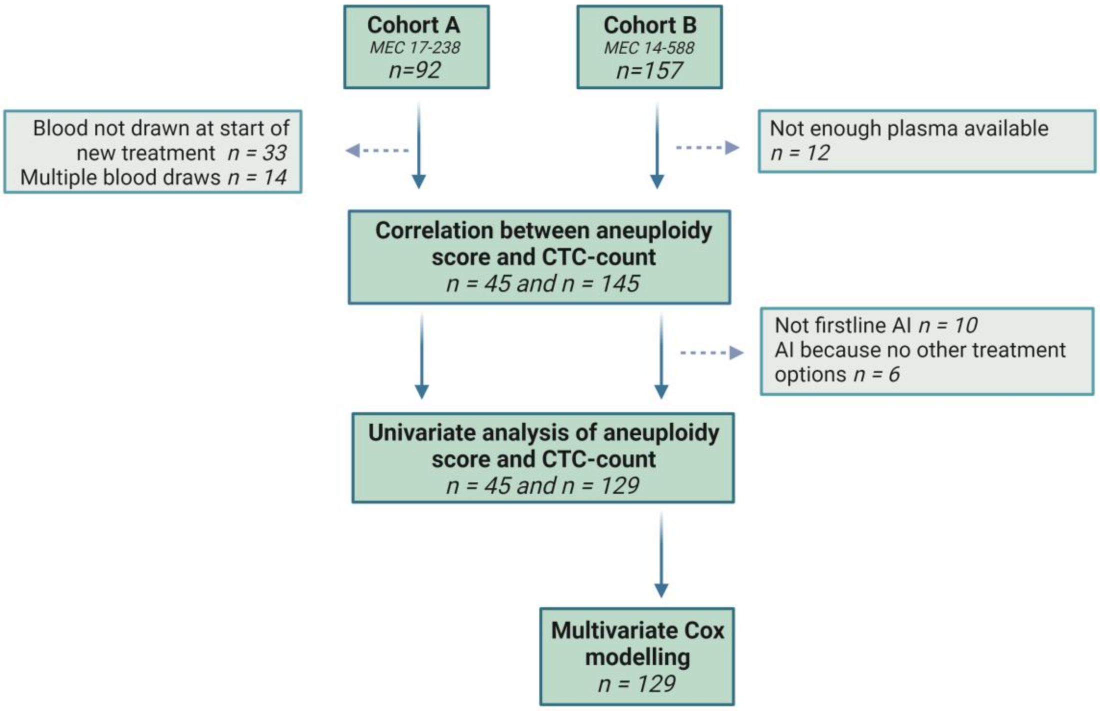

Supplementary Figure 1. Sample flow. Flowchart showing the REMARK diagram of sample selection.

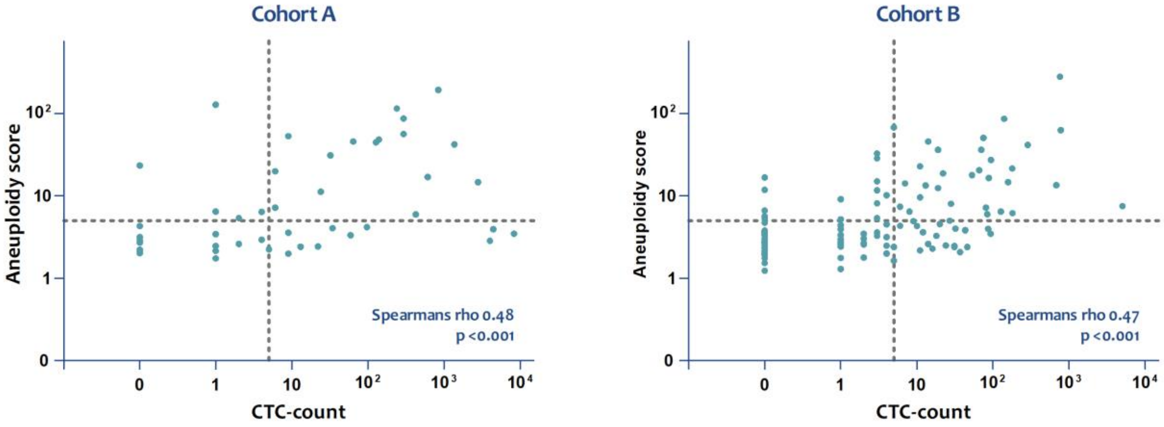

Supplementary Figure 2. Correlation between aneuploidy score and CTC-count. Scatter plots comparing aneuploidy score and CTC-count for cohort A (left) and cohort B (right). Dotted lines represent used cut-off values of five for CTC-count and five for aneuploidy score. Spearman correlation coefficients were 0.48 and 0.47, respectively (both $p < 0.001$).

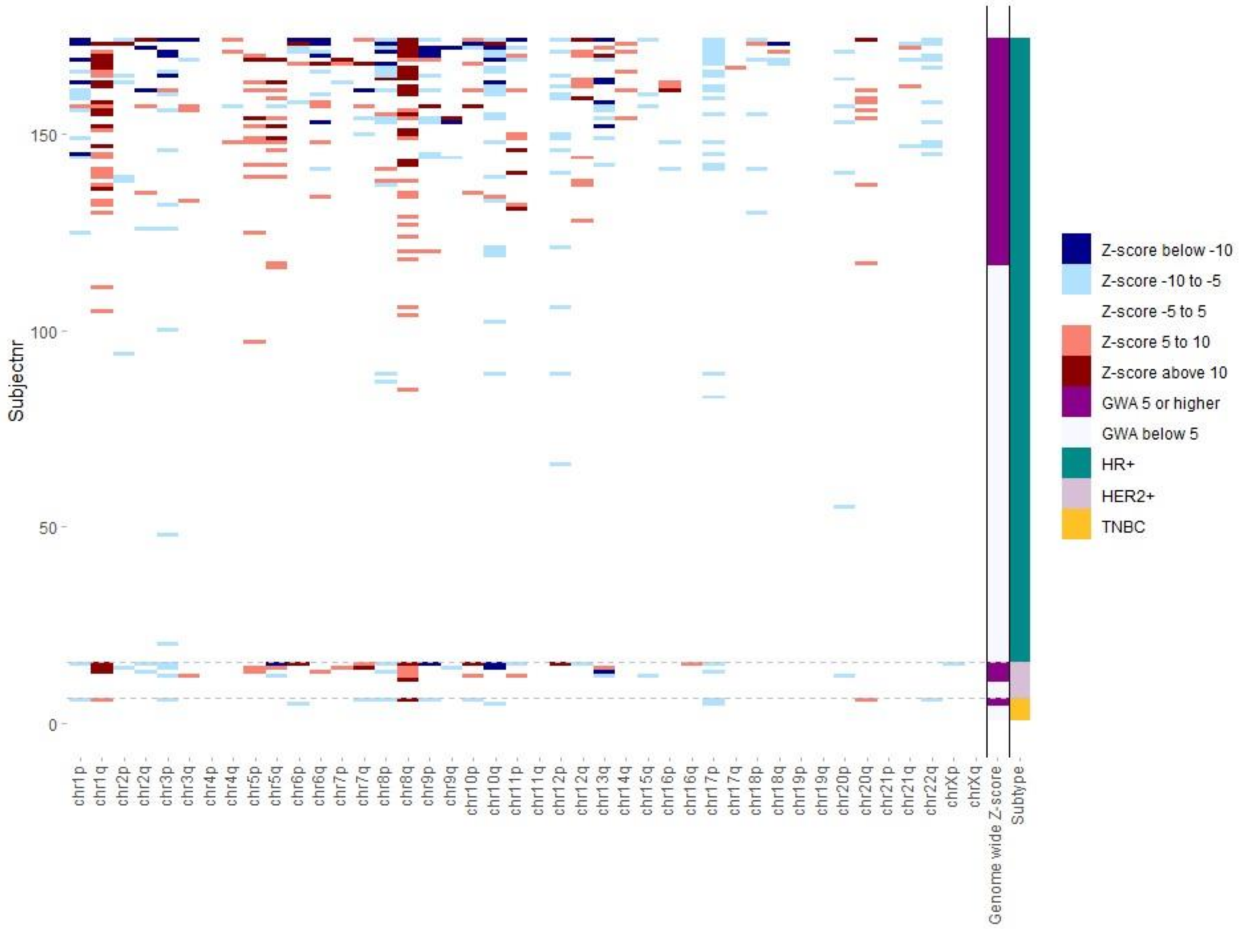

Supplementary figure 3. Detailed overview of aneuploidy score per sample. Heatmap showing z-scores per chromosome arm per subject, regions with a significant high read count compared to healthy blood donors in red and with a significant low read count in blue. The right columns show if the genome-wide aneuploidy score (GWA) was above (purple) or below (white) the used cut-off of 5, and the breast cancer subtype. Although the numbers were small, it is shown that the aneuploidy score was elevated in the HER2+ and TNBC subtypes as well.

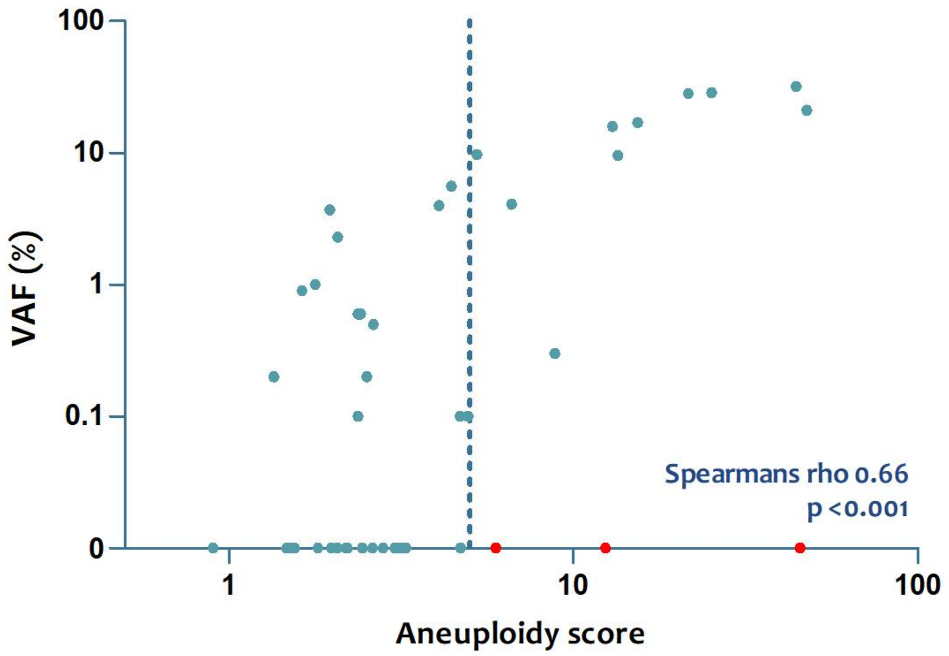

Supplementary Figure 4. Correlation between aneuploidy score and VAF. Scatter plots comparing variant allele frequency (VAF) of the dominant mutation on the Oncomine™ Breast cfDNA panel and aneuploidy score (Spearman’s correlation coefficient 0.66, p <0.001). The dotted line on the x-axis represents the aneuploidy cut-off of 5. Red dots indicate patients with no detectable mutation, but a high aneuploidy score.

Supplementary table 1. Primers for mFAST-SeqS.

LINE-1 PCR	
	sequence (5'-3')
forward	TCTTTCCCTACACGACGCTCTTCCGATCT**ACACAGGGAGGGGAACAT**
	TCTTTCCCTACACGACGCTCTTCCGATCT*A***TACACAGGGAGGGGAACAT**
	TCTTTCCCTACACGACGCTCTTCCGATCT*TC***TACACAGGGAGGGGAACAT**
	TCTTTCCCTACACGACGCTCTTCCGATCT*CTA***TACACAGGGAGGGGAACAT**
	TCTTTCCCTACACGACGCTCTTCCGATCT*GATA***TACACAGGGAGGGGAACAT**
	TCTTTCCCTACACGACGCTCTTCCGATCT*ACTCA***TACACAGGGAGGGGAACAT**
	TCTTTCCCTACACGACGCTCTTCCGATCT*TTCTCTT***ACACAGGGAGGGGAACAT**
	TCTTTCCCTACACGACGCTCTTCCGATCT*CACTTCTT***ACACAGGGAGGGGAACAT**
reverse	GTGACTGGAGTTCAGACGTGTGCTCTTCCGATCT**TGCCATGGTGGTTTGCT**
	LINE-1 specific sequences are indicated in bold. Spacers are underlined and italic

Index PCR	
Forward	AATGATACGGCGACCACCGAGATCTACAC**TCTTTCCCTACACGACGCTCTTCCGATCT**
Rev_Index1	CAAGCAGAAGACGGCATACGAGATCGTGAT**GTGACTGGAGTTCAGACGTGTGCTCTTCCGATCT**
Rev_Index2	CAAGCAGAAGACGGCATACGAGATACATCG**GTGACTGGAGTTCAGACGTGTGCTCTTCCGATCT**
Rev_Index3	CAAGCAGAAGACGGCATACGAGATGCCTAA**GTGACTGGAGTTCAGACGTGTGCTCTTCCGATCT**
Rev_Index4	CAAGCAGAAGACGGCATACGAGATTGGTCA**GTGACTGGAGTTCAGACGTGTGCTCTTCCGATCT**
Rev_Index5	CAAGCAGAAGACGGCATACGAGATCACTGT**GTGACTGGAGTTCAGACGTGTGCTCTTCCGATCT**
Rev_Index6	CAAGCAGAAGACGGCATACGAGATATTGGC**GTGACTGGAGTTCAGACGTGTGCTCTTCCGATCT**
Rev_Index7	CAAGCAGAAGACGGCATACGAGATGATCTG**GTGACTGGAGTTCAGACGTGTGCTCTTCCGATCT**
Rev_Index8	CAAGCAGAAGACGGCATACGAGATTCAAGT**GTGACTGGAGTTCAGACGTGTGCTCTTCCGATCT**
Rev_Index9	CAAGCAGAAGACGGCATACGAGATCTGATC**GTGACTGGAGTTCAGACGTGTGCTCTTCCGATCT**
Rev_Index10	CAAGCAGAAGACGGCATACGAGATAAGCTA**GTGACTGGAGTTCAGACGTGTGCTCTTCCGATCT**
Rev_Index11	CAAGCAGAAGACGGCATACGAGATGTAGCC**GTGACTGGAGTTCAGACGTGTGCTCTTCCGATCT**
Rev_Index12	CAAGCAGAAGACGGCATACGAGATTACAAG**GTGACTGGAGTTCAGACGTGTGCTCTTCCGATCT**
Rev_Index13	CAAGCAGAAGACGGCATACGAGATTTGACT**GTGACTGGAGTTCAGACGTGTGCTCTTCCGATCT**
Rev_Index14	CAAGCAGAAGACGGCATACGAGATGGAACT**GTGACTGGAGTTCAGACGTGTGCTCTTCCGATCT**
Rev_Index15	CAAGCAGAAGACGGCATACGAGATTGACAT**GTGACTGGAGTTCAGACGTGTGCTCTTCCGATCT**
Rev_Index16	CAAGCAGAAGACGGCATACGAGATGGACGG**GTGACTGGAGTTCAGACGTGTGCTCTTCCGATCT**
Rev_Index18	CAAGCAGAAGACGGCATACGAGATGCGGAC**GTGACTGGAGTTCAGACGTGTGCTCTTCCGATCT**
Rev_Index19	CAAGCAGAAGACGGCATACGAGATTTTCAC**GTGACTGGAGTTCAGACGTGTGCTCTTCCGATCT**
Rev_Index20	CAAGCAGAAGACGGCATACGAGATGGCCAC**GTGACTGGAGTTCAGACGTGTGCTCTTCCGATCT**
Rev_Index21	CAAGCAGAAGACGGCATACGAGATCGAAAC**GTGACTGGAGTTCAGACGTGTGCTCTTCCGATCT**
Rev_Index22	CAAGCAGAAGACGGCATACGAGATCGTACG**GTGACTGGAGTTCAGACGTGTGCTCTTCCGATCT**
Rev_Index23	CAAGCAGAAGACGGCATACGAGATCCACTC**GTGACTGGAGTTCAGACGTGTGCTCTTCCGATCT**
Rev_Index25	CAAGCAGAAGACGGCATACGAGATATCAGT**GTGACTGGAGTTCAGACGTGTGCTCTTCCGATCT**
Rev_Index27	CAAGCAGAAGACGGCATACGAGATAGGAAT**GTGACTGGAGTTCAGACGTGTGCTCTTCCGATCT**
	common sequence targeting the LINE-1 primers is indicated in bold
